# The Role of UPF0157 in the Folding of *M. tuberculosis* Dephosphocoenzyme A Kinase and the Regulation of the Latter by CTP

**DOI:** 10.1371/journal.pone.0007645

**Published:** 2009-10-30

**Authors:** Guneet Walia, Parimal Kumar, Avadhesha Surolia

**Affiliations:** 1 Molecular Biophysics Unit, Indian Institute of Science, Bangalore, India; 2 National Institute of Immunology, New Delhi, India; Charité-Universitätsmedizin Berlin, Germany

## Abstract

**Background:**

Targeting the biosynthetic pathway of Coenzyme A (CoA) for drug development will compromise multiple cellular functions of the tubercular pathogen simultaneously. Structural divergence in the organization of the penultimate and final enzymes of CoA biosynthesis in the host and pathogen and the differences in their regulation mark out the final enzyme, dephosphocoenzyme A kinase (CoaE) as a potential drug target.

**Methodology/Principal Findings:**

We report here a complete biochemical and biophysical characterization of the *M. tuberculosis* CoaE, an enzyme essential for the pathogen's survival, elucidating for the first time the interactions of a dephosphocoenzyme A kinase with its substrates, dephosphocoenzyme A and ATP; its product, CoA and an intrinsic yet novel inhibitor, CTP, which helps modulate the enzyme's kinetic capabilities providing interesting insights into the regulation of CoaE activity. We show that the mycobacterial enzyme is almost 21 times more catalytically proficient than its counterparts in other prokaryotes. ITC measurements illustrate that the enzyme follows an ordered mechanism of substrate addition with DCoA as the leading substrate and ATP following in tow. Kinetic and ITC experiments demonstrate that though CTP binds strongly to the enzyme, it is unable to participate in DCoA phosphorylation. We report that CTP actually inhibits the enzyme by decreasing its Vmax. Not surprisingly, a structural homology search for the modeled mycobacterial CoaE picks up cytidylmonophosphate kinases, deoxycytidine kinases, and cytidylate kinases as close homologs. Docking of DCoA and CTP to CoaE shows that both ligands bind at the same site, their interactions being stabilized by 26 and 28 hydrogen bonds respectively. We have also assigned a role for the universal Unknown Protein Family 0157 (UPF0157) domain in the mycobacterial CoaE in the proper folding of the full length enzyme.

**Conclusions/Significance:**

In view of the evidence presented, it is imperative to assign a greater role to the last enzyme of Coenzyme A biosynthesis in metabolite flow regulation through this critical biosynthetic pathway.

## Introduction

*Mycobacterium tuberculosis*, the causative organism of tuberculosis, possesses a thick, hydrophobic, complex cell envelope composed of a variety of complex lipids, peptidoglycans etc. which serves as an impervious barrier to drugs [1]–[3]. An excellent means to combat the pathogen is to target biosynthetic pathways of vitamins and cofactors which are indispensable in varied cellular functions [4], [5]. One such ubiquitous cofactor is Coenzyme A (CoA) which along with its precursor, 4′-phosphopantetheine, functions as an acyl group carrier and carbonyl activating group for Claisen reactions as well as for amide-, ester-, and thioester-forming reactions in the cell acting as prosthetic groups of carrier proteins of fatty acid, polyketide, nonribosomal peptide synthases etc. [6]. A survey of the BRENDA database (http://www.brenda-enzymes.info) of all known enzyme activities shows that 9% of the approximately 3500 identified activities use CoA or a CoA thioester as a co-substrate and therefore, the ratio between CoA and its thioester derivatives is important for maintaining cellular homeostasis [7]. This is further corroborated by the fact that all attempts to disrupt the genes coding for the enzymes in the CoA biosynthetic pathway have either failed or have resulted in lethal phenotypes in many organisms.

CoA biosynthesis, a five-step pathway which utilizes the precursors, pantothenate, cysteine and ATP, is invariant in all organisms [6]. It begins with the phosphorylation of pantothenate by the enzyme CoaA (pantothenate kinase). CoaB (phosphopantethenoylcysteine synthetase) condenses the 4′-phosphopantothenate thus generated with a cysteine molecule to form 4′-phosphopantothenoylcysteine which is further decarboxylated by CoaC (phosphopantothenoylcysteine decarboxylase) liberating 4′-phosphopantetheine, the prosthetic group incorporated by carrier proteins in fatty acid, polyketide, nonribosomal peptide biosyntheses. CoaD (phosphopantetheine adenylyltransferase) adenylates the 4′-phosphopantetheine moiety to generate the final CoA precursor, dephosphocoenzyme A (DCoA). CoaE (dephosphocoenzyme A kinase, EC 2.7.1.24) then phosphorylates the 3′-OH of the ribose in the DCoA structure to generate CoA.

In mammals, the activities of the penultimate enzyme CoaD and the final enzyme CoaE are carried out by a single bifunctional 62 kDa enzyme, encoded by the CoA synthase gene [8], [9]. The human and porcine CoA synthases have been shown to possess three domains, an N-terminal domain that helps target the bifunctional enzyme to the mitochondrial outer membrane and mediates protein-protein interactions, a middle CoaD domain and a C-terminal CoaE domain [10]. Interestingly, it has been shown that proteolytic cleavage of the human enzyme does not affect enzyme activity or the proposed tertiary structure [11]. Also, the N-terminal domain of the CoA synthase is found only in higher eukaryotes and has been shown to effect regulation of the CoaD and CoaE activities by phospholipids [12]. The amalgamation of the last two enzyme activities also hints at the existence of a currently-unknown, relatively recently evolved mechanism of regulation of the CoA biosynthetic pathway seen only in the higher eukaryotes. The mycobacterial enzyme shows a very low sequence similarity (15%) to the human CoaE. Thus low sequence homology and different mechanisms of regulation in the prokaryotic and eukaryotic sequences show that selective inhibition of the mycobacterial CoA biosynthesis is possible, marking out dephosphocoenzyme A kinase (CoaE) as a potential antimycobacterial drug target.

Unlike the first step of mycobacterial CoA biosynthesis [13]–[15], information about its last step, catalyzed by CoaE, is scanty. Here we report a complete biochemical and biophysical characterization of the putative *M. tuberculosis* dephosphocoenzyme A kinase (Rv1631) detailing the kinetic parameters of catalysis, the energetics and order of substrate binding to the enzyme and the phosphate donor specificity of the kinase. We show that its activity is regulated by an intrinsic cellular metabolite, cytidine triphosphate (CTP) a feature as yet unknown for members of this family of enzymes. The mycobacterial enzyme is atypical compared to its counterparts in the prokaryotic kingdom as it possesses an extra domain C-terminal to the dephosphocoenzyme A kinase, which belongs to the Unknown Protein Family, UPF0157 (Pfam database); to which no function has been assigned till date. We show that this domain is essentially required for the proper folding of the mycobacterial CoaE, delineating region 35–50 (KIACGHKALRVDHIG) of this domain as essential for the latter function.

## Materials and Methods

### Plasmid Construction, Expression and Purification of CoaE

The full length CoaE gene was amplified from *M. tuberculosis* genomic DNA using the primers CoaE_Fwd:5′- GGAATTCCATATGGTGACCGACCGCGAT–3′ and CoaE_Rev:5′- CCCAAGCTTTTAACGCAAATGCAC-3′, designed with NdeI/HindIII sites. The resulting amplification product was subcloned into the NdeI/HindIII sites of the pET28a+ expression vector for recombinant expression in *E. coli* BL21 (DE3) competent cells. Sequencing the putative clone using T7 promoter and terminator helped confirm the final clone. For optimal yield of the hexahistidine-tagged recombinant protein, cells were grown at 37°C to an O.D_600∼_0.7 and induced with 0.1 mM isopropyl-1-thio-β-D-galactopyranoside (IPTG) for 8 h at 18°C. The cells were harvested, resuspended in binding buffer (10 mM Tris-HCl, 500 mM NaCl, 10% glycerol, pH 7.8) and lysed by sonication twice for 10 min on ice. The cell free lysate was loaded onto charged Ni- NTA affinity resin (Novagen) and eluted with 100 mM imidazole. The eluted protein was dialyzed against standard buffer (10 mM Tris, 150 mM NaCl, 5% glycerol, pH-7.8) and concentrated.

### Enzyme Assays

All enzyme assays were carried out at 25°C. Units of activity correspond to 1 umol of product formed/min, and any blank rates without DCoA, taken as controls, were subtracted. CoaE activity was assayed using two different coupled assays; the established double coupled pyruvate kinase/lactate dehydrogenase (PK/LDH) and a single-coupled α-ketoglutarate dehydrogenase (KDH) assay [16]. The latter assays for CoA formation by monitoring an increase in absorbance at 340 nm due to NADP+ reduction. Each reaction mixture for the α-KDH assay contained ATP (10 mM), DCoA (0.5 mM), MgCl_2_ (10 mM), KCl (20 mM), α-ketoglutarate dehydrogenase (2 U), α-ketoglutarate (2 mM), NADP (0.3 mM), thiamine pyrophosphate (0.3 mM) in 50 mM Tris, pH 8.0. Enzyme activity was further confirmed by separating the products of the reaction mixture containing DCoA (0.25 mM), ATP (0.25 mM), γP^32^-ATP (0.5 uCi), MgCl_2_ (10 mM), DTT (1.5 mM) in 50 mM Tris, pH 8.0, by descending paper chromatography on a Whatman 3 MM paper in a developing system containing isobutyric acid:0.5 N ammonium hydroxide (100∶60) and 1 mM EDTA.

### ITC Experiments

Since we assayed the enzyme using coupled assays which could be prone to errors that could account for the high affinity of the mycobacterial enzyme for its substrates and the higher catalytic efficiency, compared to its other prokaryotic counterparts, we chose to quantify the kinetic parameters also using a direct, thermodynamic approach (Table 1). A thermodynamically favorable reaction is accompanied by a decrease in free energy (−ΔG), which is composed of enthalpic and entropic terms (ΔG = ΔH−TΔS). Calorimetry measures the heat exchanged during any reaction [17]. The high sensitivity of the technique allows experiments to be carried out with low amounts of enzyme and a single experiment allows rate calculations multiple times as several injections of the substrate into the cell containing the enzyme can be carried out. ITC experiments were performed using a VP-ITC titration microcalorimeter (Microcal Inc., U.S.A.). The reference cell was filled with water and the calorimeter was calibrated using standard electrical pulses as recommended by the manufacturer. For all ITC experiments, the CoaE protein was dialyzed overnight (12–18 hr) in 10 mM Tris buffer, pH 7.8, 150 mM NaCl, 10 mM MgCl_2_, 20 mM KCl and 5% glycerol. Substrate solutions were prepared in the final dialysis buffer. Solutions of the protein were filled in the sample cell (1.4 ml) and titrated with ATP-γS (a non-hydrolysable analog of ATP) or DCoA solutions introduced into it from the syringe (298 uL). Excepting the substrate against which the parameters were being determined, the composition of the solutions in the syringe and cell were kept constant. ITC experiments were routinely performed at 20°C. Raw data were collected for substrate heats of dilution in the buffer and integrated using the Microcal Origin software version 7.0 supplied with the instrument. In control experiments, the substrates were titrated against buffer.

**Table 1:** Thermodynamic parameters of ligand binding to mycobacterial CoaE at pH 7.8 and 20°C.

Titrant	*N*	*ΔH (cal/M^−1^)*	K_a_ (M^−1^*104)	*ΔS (calM^−1^K^−1^)*
ATP alone	0.683	−495.3 (±36.3)	1.18 (±0.14)	16.9 (±1.21)
DCoA alone	0.810	−3719 (±201.5)	1.43 (±0.34)	6.32 (±0.34)
NHATP binding on DCoA-saturated CoaE	0.947	−3776 (±234.5)	2.06 (±0.47)	6.85 (±0.48)
DCoA binding on NH-ATP-saturated CoaE	0.839	−1063 (±71.3)	1.85 (±0.25)	15.9 (±0.91)
CoA alone	NB	NB	NB	NB
ADP binding on DCoA-saturated CoaE	1.07	−3612 (274.8)	2.03 (±0.51)	7.38 (±0.78)
ADP binding on CoA-saturated CoaE	1.23	−2138 (±181.5)	2.88 (±0.58)	13.1 (±0.98)
dATP binding on DCoA-saturated CoaE	0.871	−848 (±54.7)	1.74 (±0.24)	16.4 (±1.85)
CTP binding	1.08	−482.5 (±37.4)	1.59 (±0.19)	17.6 (±1.11)
GTP binding	NB	NB	NB	NB

Values of ΔH and ΔS are in cal mol^−1^K^−1^. Values are the mean of five individual experiments. K_a_ is the binding constant determined by ITC and its values are in M^−1^.

### Determination of the Kinetic Parameters of ATP Hydrolysis by ITC

To evaluate the kinetic parameters of ATP hydrolysis, 1 µM of dialyzed protein was incubated with 1 mM of DCoA for 45 mins on an end-on rocker at 4°C for saturation. The reaction was started by injecting 20 µl of the 1 mM ATP solution into the sample cell loaded with DCoA-saturated CoaE. A single injection of 1 mM ATP (20 µl), prepared in the final dialysis buffer, was monitored for 3000 seconds. Heat change during the course of reaction was used to determine the ΔH for the reaction. To determine the kinetic constants, the protocol of ligand-multiple injections was adopted, which also helped ascertain the maximum velocity for the enzymatic reaction [17]. A gap of 110 seconds was kept between subsequent injections. Therefore, change in power (Δ*P*) with respect to the added ligand helped determine the kinetic constants for ATP hydrolysis using following two equations.
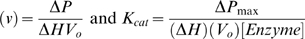
where Δ*P* is the instant power generated by the reaction, Δ*P_max_* is the maximum power generated, Δ*H* is the enthalpy change of the reaction and *V_o_* is the volume of the ITC cell, [Enzyme] is the concentration of enzyme present in the ITC cell [17]. Kinetic parameters with respect to DCoA were determined in a similar fashion as those for ATP.

### Kinetics of the Reverse Reaction

In order to determine the kinetic parameters of the reverse reaction the enzyme was saturated with 1 mM ADP and titrated against 1 mM CoA. In another set of reactions, the enzyme was saturated with 1 mM CoA and titrated against 1 mM ADP. For both the sets of reactions, single as well as multiple injection runs were carried out as above.

### ITC Studies for Substrate Binding to CoaE

Binding studies were carried out with DCoA, CoA, ATP-γS and ADP. The sample cell was filled with 200 uM CoaE. For each binding experiment with one substrate, 1 mM solution of the ligand was introduced from the syringe into the cell containing the enzyme saturated with the other substrate. For this purpose the enzyme was incubated with the substrate to be saturated with, on an end-on rocker for 45 mins, at 4°C. Titrations were carried out by the stepwise addition of small volumes (10 ul) of the ligand solution from the constantly stirring syringe (286 rpm) into the CoaE containing sample cell with a time interval of 180 seconds between successive injections. Change in the enthalpy (ΔH), K_b_ and *n* values for the titration were determined by non-linear least squares fit of the data using Origin Tm 7.0 software. The data were fitted to a single-site binding model by a non-linear regression analysis to yield binding constants (*K*_a_), enthalpies of binding (**Δ** *H*) and the stoichiometry of binding (*N*).

### Mycobacterial CoaE Homology Modeling Studies

No structures are currently available for CoaEs fused to another domain. In order to aid the selection of modeling templates for the N- and C-terminal domains of the mycobacterial CoaE, the Sequence Feature Scan tool from the Swiss-Model was used (Supporting Information File S1, Table S2) [18]. Putative models for the NTD and CTD were obtained by the SWISS-MODEL software of the SWISSPROT workspace using both the first approach mode as well as the alignment interface mode. Since the closest homolog, with a solved structure, to the mycobacterial enzyme, was the *E. coli* CoaE, we modeled the NTD on the *E. coli* structure (32.7% identity, 54.7% similarity). In 2006, a crystal structure for the GrpB protein of unknown function from *Enterococcus faecalis* was deposited in the PDB, (PDB code: 2nrk, accession no. Q837C3_ENTFA) [19]. This protein also belongs to the UPF0157 family of proteins of unknown function like the CTD of the mycobacterial CoaE, with which it shares a sequence identity of 25.5%. No other crystal structures were available for this domain except the 2nrk structure and as the CTD shares considerable sequence similarity with the enterococcal protein (e-value of 4e-48 in PSI-BLAST search), the latter was used as a template to model the CTD using the Swiss-Model software in the alignment interface mode. The models of the NTD and CTD thus obtained were then energy minimized using the Molecular Operating Environment (MOE) utility, version MOE2006.08, using the MMFF94x forcefield and were then stitched together to generate the modeled mycobacterial CoaE by the Coot software [20], [21]. We extensively evaluated the model quality using different software (Anolea, GROMOS, WhatCheck, PROCHECK and ProQres, Supporting Information File S1). The RMS Z-scores from WhatCheck showed that the model obtained was acceptable and the Ramachandran plot showed that the overall conformation of the backbone of the model was reliable. Fig. S3 shows that the predicted model accuracy scores lay well within the acceptable range.

### Ligand Docking

Ligands used for docking were built using both MOE as well as the ChemDraw Ultra Version 5.0 software. These were energy minimized by MOE using the AMBER99 forcefield and then docked onto the energy minimized protein models using the MMFF94x forcefield in the MOE server. The docked complex with the least energy was chosen. Each process starting from model building, energy minimization and ligand docking was carried out a minimum of 15 times for each ligand-protein complex. A detailed analysis of the interatomic contacts, interface complimentarity for each ligand-protein complex and a putative list of hydrogen bonds was calculated using the LPC/CSU software (Tables 2 and 3, Supporting Information File S1) [22].

**Table 2:** Types of contacts made by the modeled mycobacterial CoaE with its substrate, Dephosphocoenzyme A, its product Coenzyme A and the metabolic regulator, CTP, as calculated by the LPC/CSU software [22].

Types of Contacts	Dephosphocoenzyme A	Coenzyme A	CTP
Hydrogen bonds	26	24	28
Hydrophobic	20	19	13
Aromatic-Aromatic	4	-	2
Acceptor-Acceptor	2	3	1
Other	37	51	41

**Table 3:** Analysis of the docked ligands.

	Dephosphocoenzyme A			Coenzyme A			CTP		
Complex Surf(A^2^)	453.1			529.8			88.9		
Uncomplex Surf(A^2^)	890.1			996.7			612		
Interact. Residue	Dist(Å)	Surf(Å^2^)	No. of contacts	Dist(Å)	Surf(Å^2^)	No. of contacts	Dist(Å)	Surf(Å^2^)	No. of contacts
**GLY 9**	-	-	-	3.8	4.9	3	-	-	-
**ILE 10**	5	25	6	4.3	22.6	10	-	-	-
**GLY 11**	-	-	-	3.7	42.6	5	-	-	-
**ALA 12**	-	-	-	4.6	0.2	1	-	-	-
**GLY 13**	-	-	-	3.8	3.1	2	-	-	-
**LYS 14**	-	-	-	3.4	58.4	11	-	-	-
**SER 15**	-	-	-	3.8	29.1	7	-	-	-
**LEU 16**	-	-	-	4.6	9.2	2	-	-	-
**GLY 31**	5	2.7	2	-	-	-	-	-	-
**ASP 32**	2.4	50.4	15	5.4	2.9	1	2.4	49.9	4
**ALA 35**	3.9	27.6	5	-	-	-	3.4	14.3	2
**ARG 36**	2.9	32.8	9	2.6	64.6	12	3.3	24.6	10
**VAL 39**	3.6	15.4	1				3.6	15.4	1
**GLN 40**	-	-	-	6	5.4	2	-	-	-
**LEU 58**	-	-	-	5.8	0.6	1	-	-	-
**LEU 64**	-	-	-	5.3	7.6	2	-	-	-
**ASP 65**	-	-	-′	3.6	28.9	7	-	-	-
**ARG 66**	2.5	126.5	16	2.5	116.1	21	3	60.2	13
**GLN 67**	3.4	33.2	7	3.8	46	15	-	-	-
**LEU 69**	-	-	-	-	-	-	3.2	22.3	5
**ALA 70**	-	-	-	-	-	-	4.4	5.4	2
**ALA 73**	-	-	-	-	-	-	3.6	42.1	6
**PHE 74**	-	-	-	-	-	-	3.8	47.3	9
**ARG 80**	-	-	-	-	-	-	2.7	63.4	10
**LEU 83**	-	-	-	-	-	-	3.3	14.3	4
**ASN 84**	-	-	-	-	-	-	3.9	44.9	8
**VAL 87**	4.6	17	3	-	-	-	3.6	30.2	8
**HIS 88**	3.7	28.2	14	-	-	-	5	12	10
**VAL 91**	5	4.9	1	-	-	-	-	-	-
**ARG 95**	6.2	0.4	1	-	-	-	-	-	-
**ILE 112**	3.8	50.4	16	-	-	-	3.7	17.7	1
**PRO 113**	4	9.4	2	-	-	-	-	-	-
**VAL 116**	-	-	-	-	-	-	4.7	0.7	1
**GLU 117**	2.6	42	4	-	-	-	2.2	44.5	5
**ARG 153**	5.3	5	2	-	-	-	-	-	-
**ALA 154**	5.6	6.6	2	-	-	-	-	-	-
**ALA 157**	3.9	28.6	7	-	-	-	2.4	46.8	8
**ALA 158**	3.5	25.3	2	-	-	-	4	11.3	1
**ARG 140**	-	-	-	3.8	56.6	12	-	-	-
**GLN 144**	-	-	-	4.8	4.7	3	-	-	-

The first two lines show the solvent-accessible surface area (Å^2^) of the ligands (the enzyme's substrate, dephosphocoenzyme A; its product, coenzyme A and its metabolic regulator, CTP) in complex with the protein and in their uncomplexed forms. Then follows a detailed comparative analysis of the residues in the mycobacterial CoaE interacting with the ligands, showing the distance between the interacting residue and the ligand in Å, the surface area of contact in Å^2^ and the number of contacts formed by an individual amino acid residue with the ligand.

### Cloning, Expression, and Purification of the N-terminal Domain

The N-terminal 196 amino acid long dephosphocoenzyme A kinase domain of the mycobacterial CoaE was cloned from the *M. tuberculosis* genomic DNA using the primers CoaE_Fwd:5′- GGAATTCCATATGGTGACCGACCGCGAT–3′and NTD-Rev: 5′-CCCAAGCTTTTAGGGCTGGACGCGCGTGTTCCAGACG-3′ designed to ligate in the NdeI/HindIII sites of pET 28a+ expression vector to obtain a hexa-histidine-tagged recombinant protein which was expressed in *E. coli* BL21 (DE3) cells. When conditions similar to those used for the expression and purification of the full-length enzyme did not yield soluble NTD, variations in temperature post induction, concentration of IPTG for induction and total culture volume were implemented. Though soluble expression of the His-tagged NTD increased very slightly using 12-litre cultures, the now-relatively-soluble hexahistidine tagged protein did not bind to the Ni-NTA column possibly due to an altered pattern of folding in the absence of the CTD, which possibly caused the hexa-histidine tag to get buried and therefore unavailable for binding to the column. We then resorted to classical protein purification methods like urea denaturation and ammonium sulphate precipitation to increase yield but none of the methods yielded sufficient quantities of soluble protein [23]. The NTD was then cloned in the pETGEXCT vector and this GST-tagged protein was then loaded on a Glutathione-Agarose column and eluted using reduced glutathione.

### Cloning, Expression, and Purification of the C-terminal Domain and Its Various Deletes

The 211-amino acid long UPF0157 domain of the mycobacterial CoaE was cloned in the pET28a+ vector in the NdeI/HindIII sites using the primers CTD-For: 5′-GGAATTCCATATGGCGCACAA-3′ and CoaE_Rev: 5′-CCCAAGCTTTTAACGCAAATGCAC-3′. Primer sequences for all the CTD deletes are as mentioned in Table S1. These histidine-tagged proteins were expressed and purified using conditions similar to those used for the full-length enzyme.

## Results

**Figure 1:**
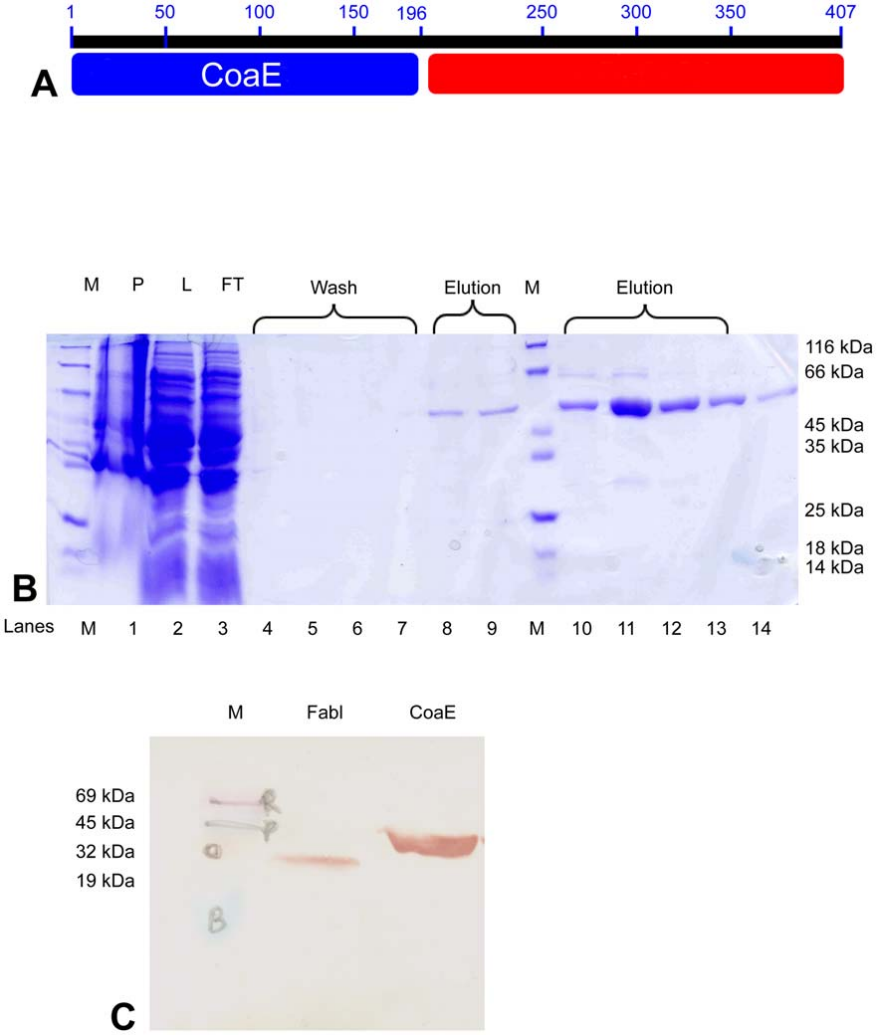
Mycobacterial CoaE domain organization and protein expression. *A*, The mycobacterial CoaE has a 196 amino acid (a.a.) N-terminal dephosphocoenzyme A kinase and a 211 a.a. C-terminal domain of unknown function, the UPF0157. *B*, elution profile of hexahistidine tagged mycobacterial CoaE purified by affinity chromatography as collected fractions loaded on a 12% SDS-PAGE, M, protein molecular weight marker; P, cell pellet obtained post sonication and centrifugation; L, supernatant obtained post centrifugation of the sonicate, loaded onto the column; FT, Flow through from the column upon loading the supernatant; Wash, Fractions collected during column washing with resuspension buffer; Elution, the elution profile represented by fractions collected during elution with buffer containing 100 mM imidazole. *C*, confirmation of the clone by Western blotting using anti-Histidine antibodies against the recombinant His-tagged CoaE (Lane 3) and His-tagged *Plasmodium falciparum* FabI as a positive control (Lane2). Lane 1 carries the pre-stained molecular weight marker.

**Figure 2:**
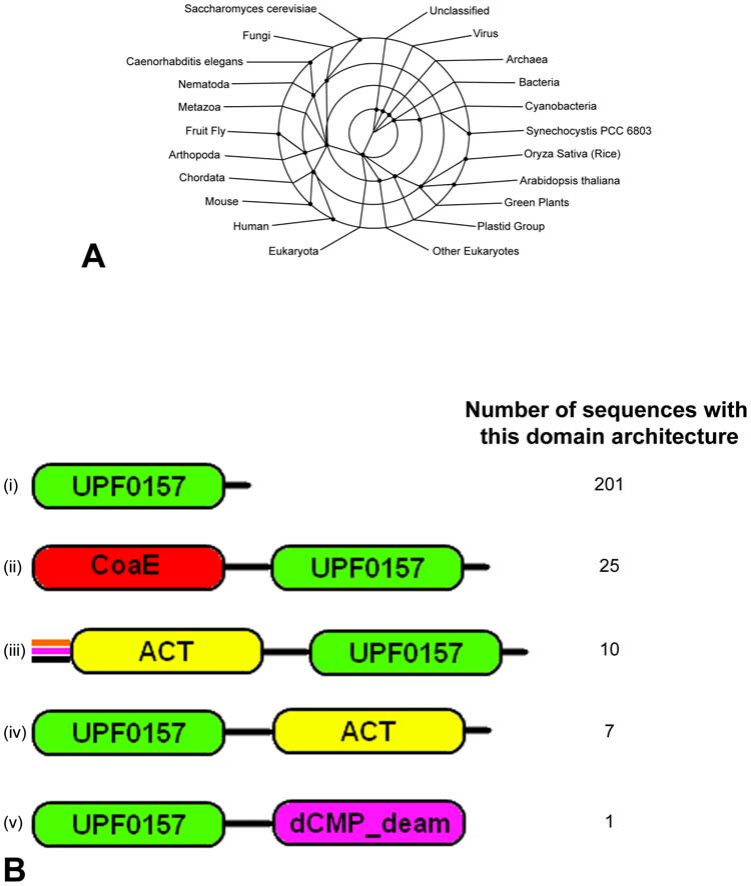
The C-terminal Domain of the mycobacterial CoaE belongs to the UPF0157 family. *A*, Taxonomic distribution of the UPF0157 proteins. *B*, The unique domain organizations/architectures in which the UPF0157 domain is found. (i) 201 sequences with the architecture: UPF0157 (ii) 25 sequences with the architecture: CoaE-UPF0157 (iii) 10 sequences with the architecture: Acetyltransferase(ACT)-UPF0157. (iv) 7 sequences with the architecture: UPF0157-Acetyltransferase (ACT) (v) one sequence with the architecture: UPF0157-cytidine and deoxycytidylate deaminase (dCMP_deam) zinc-binding region.

A BLAST analysis of the putative tubercular dephosphocoenzyme A kinase, Rv1631, reveals that the mycobacterial enzyme is a two-domain protein, with the 196-residue N-terminal domain (NTD) encoding the dephosphocoenzyme A kinase and a 211-residue C-terminal domain (CTD) belonging to the UPF0157 family of Proteins of Unknown Function also known as GrpB (Glutamate rich protein B) (Fig. 1A). The Pfam database for this family, UPF0157, shows that it is present in 174 species across all kingdoms of life, ranging from archaea, cyanobacteria, viruses, *S. cerevisiae*, *C. elegans*, the nematodes, fruit flies, mice and even humans, in 5 different domain architectures and in 244 different sequences (Fig. 2A and B). Computational analysis of Rv1631, a 1224 bp gene, showed that it does not cluster with the genes for the other enzymes of the CoA biosynthesis pathway on the mycobacterial genome. Most of the genes in the region of the genome around the *coaE* gene (1825.6 Kb to 1845.6 Kb) either code for conserved hypothetical proteins or those for which only putative functions are known till date. We cloned the mycobacterial CoaE gene in the pET28a+ vector and expressed it in *E. coli* BL21 (DE3) cells. The recombinant protein was purified by immobilized metal affinity chromatography (Fig. 1B). The expression of the hexahistidine-tagged, 47 kDa recombinant mycobacterial CoaE was further confirmed by Western blotting with anti-His antibodies (Fig. 1C).

### Characterization of CoaE

**Figure 3:**
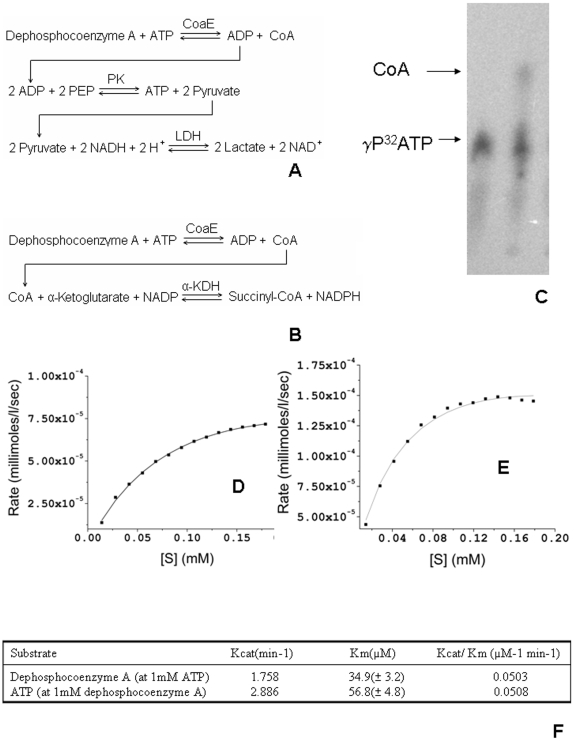
Biochemical characterization. Coupled assay systems used to determine CoaE activity. *A*, The double-coupled assay system using Pyruvate Kinase (PK) and Lactate Dehyrdrogenase (LDH) which assays for ADP formation. *B*, The single-coupled assay system using α–ketoglutarate dehydrgenase (α-KDH) which assays for CoA formation. *C*, Radioassay employing γP^32^-ATP as a substrate for CoaE. TLC shows the separation of the products of the reaction mixture containing DCoA (0.25 mM), ATP (0.25 mM), γP^32^-ATP (0.5 uCi), MgCl_2_ (10 mM), DTT (1.5 mM) in 50 mM Tris, pH 8.0, on a Whatman 3 MM paper in a developing system containing isobutyric acid:0.5 N ammonium hydroxide (100∶60) and 1 mM EDTA confirmed that the recombinant CoaE was an active enzyme. *D*, Enzyme kinetics with varying [DCoA]. *E*, Enzyme kinetics with varying [ATP]. 1.4 mL of 1 uM CoaE (dialyzed 12–18 hrs in 25 mM Tris buffer, pH 7.8, 150 mM NaCl, 10 mM MgCl_2_, 20 mM KCl, 5% glycerol) was preincubated with 1 mM DCoA for 30 mins and was titrated against 1 mM NH-ATP (298 uL) at 20°C. Raw data were collected for substrate heats of dilution in the buffer and integrated using the Microcal Origin 7.0 software. *F*, Kinetic parameters determined for the substrates of the mycobacterial CoaE, ATP and DCoA.

CoaE phosphorylates DCoA using ATP as the phosphate donor, generating CoA and ADP. Enzymatic activity was assayed using two different coupled assays, the pyruvate kinase/lactate dehydrogenase system (Fig. 3*A*) that assays for ADP formation and the α-ketoglutarate dehydrogenase single coupled system (Fig. 3*B*) that assays for CoA formation. The reaction system using γ-labeled ATP further confirmed that the hexahistidine tagged, mycobacterial CoaE, was an active protein (Fig. 3*C*). Using the coupled assays, the Km and Kcat values for ATP were found to be 56.8 µM and 2.86 min^−1^ while those for DCoA were determined as 34.9 µM and 1.75 min^−1^. Both substrates behaved as Michaelis-Menten substrates over the range of concentrations studied. Km values have only been reported from two prokaryotic sources till date, the *E. coli* enzyme (740 µM for ATP and 140 µM for DCoA) and the *Corynebacterium ammoniagenes* enzyme (120 µM for DCoA) [16], [24]. Comparison with these values shows that the mycobacterial enzyme has a higher affinity for both its substrates. Moreover, the mycobacterial CoaE is catalytically 21 times more proficient (Kcat/Km 0.0503 µM^−1^ min^−1^) as compared to its *E. coli* counterpart.

### Enzyme Assay by Isothermal Titration Calorimetry

**Figure 4:**
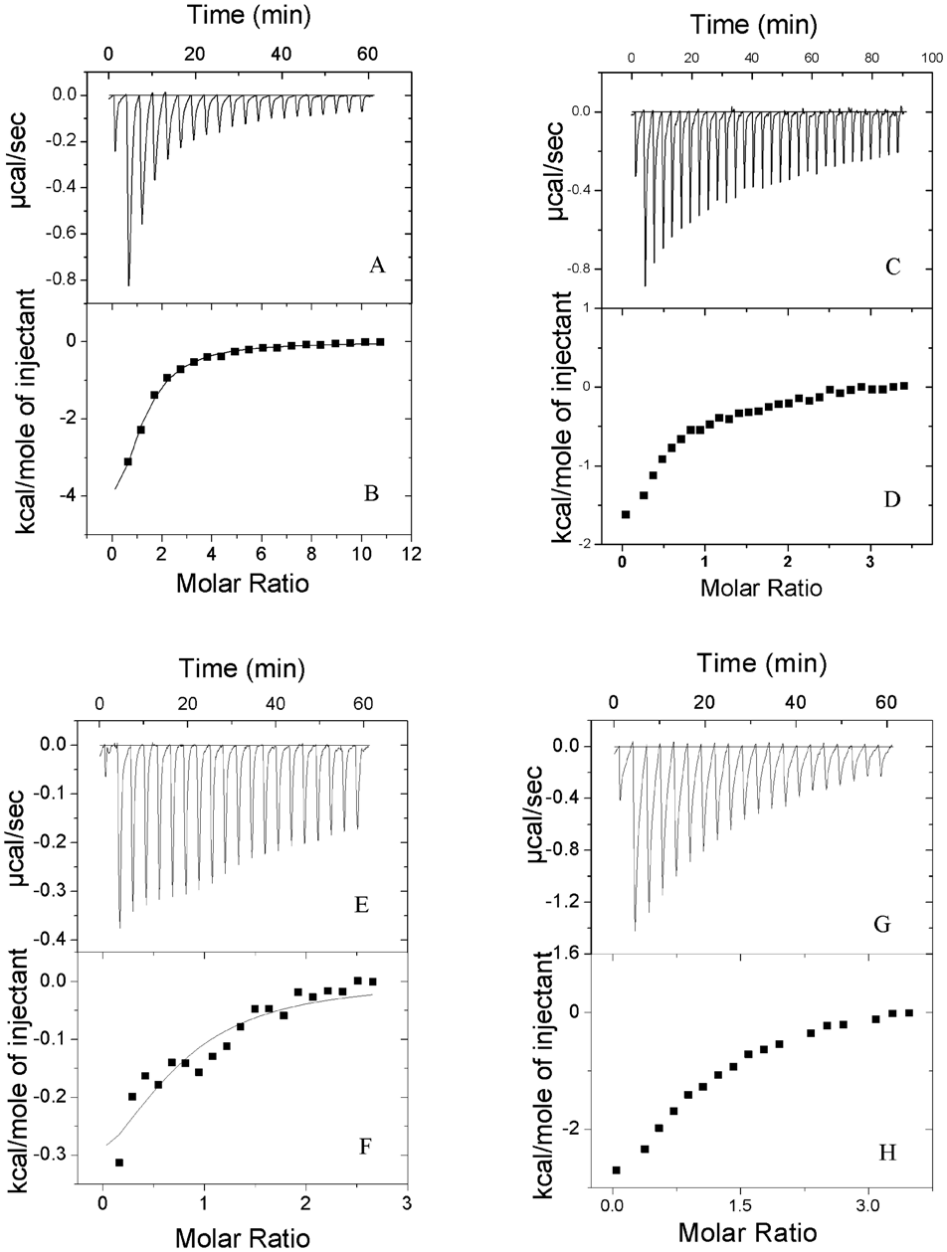
Representative ITC measurements. *A* and *B*, titration of 1 mM DCoA against 200 uM CoaE, *C* and *D*, titration of 1 mM DCoA against 1 mM ATP-saturated CoaE, *E* and *F*, titration of 1 mM ATP against 200 uM CoaE, *G* and *H*, titration of 1 mM ATP against 1 mM DCoA-saturated CoaE. Raw data are shown as differential power signals in *A*, *C*, *E*, and *G*. In *B*, *D*, *F*, and *H*, the area under the curve produced on each injection was integrated (*filled squares*) and was plotted against the molar ratio of DCoA to enzyme binding sites using the Origin 7.0 software. The *solid lines* represent nonlinear best fits for a single-site binding model. Titrations were performed in 50 mM Tris/HCl, 150 mM NaCl, 5% Glycerol, pH 7.8 at 20°C.

In order to rule out the over-estimation of kinetic constants by the coupled assays, we also adopted a more direct, thermodynamic approach to calculate the same. The Km and Kcat values calculated by ITC correlate well with those calculated by the single- and double-coupled assays (Fig. 3*D*–*F*). The negative deflections in the binding thermograms show that the reactions are exothermic (Fig. 4A, C and E). Taken together, these data reveal that the mycobacterial CoaE indeed has a higher affinity for both its substrates and a considerably greater value of Kcat (21 times) compared to its counterparts in other prokaryotes.

### Substrate Binding Studies

CoaEs belong to the P-loop containing nucleotide triphosphate hydrolase superfamily. Our ITC studies on the mycobacterial CoaE reveal as yet unknown features for this class of enzymes. The two substrates, DCoA and ATP, are presumably located in separate and well defined binding pockets of the enzyme, as is seen in all the other enzymes belonging to this superfamily of P-loop containing proteins. The ternary complex between the enzyme and its substrates may either be formed via an obligatory sequential pathway or by a random mechanism of substrate addition. Sequential addition of substrates can follow either of the following two routes: CoaE → CoaE·DCoA → CoaE·DCoA·ATP (reactions i and ii of Scheme 1) or CoaE → CoaE·ATP → CoaE·ATP·DCoA (reactions iii and iv depicted in scheme 1). On the other hand, if substrate binding follows a random mechanism, binding of one substrate to the enzyme would not be a prerequisite for binding of the other, and all four reactions of Scheme 1 can take place with equal probability.
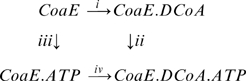



#### Scheme 1

Formation of the ternary enzyme-substrate complex, CoaE·DCoA·ATP. If the reaction follows an ordered sequential pathway, it may take the routes i and ii OR iii and iv. If, on the other hand, the substrates bind in a random fashion, all four reactions have an equal probability of occurrence.

**Figure 5:**
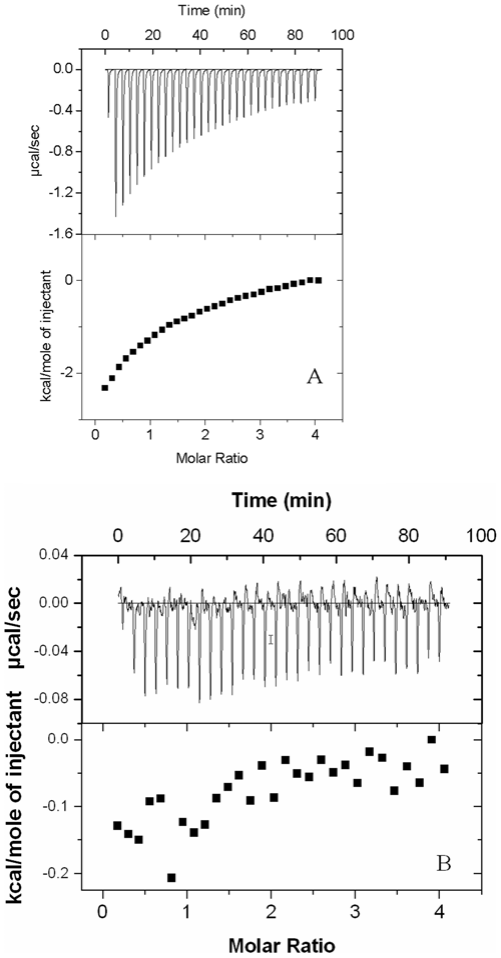
Feedback inhibition by the reaction products. *A*, 1 mM ADP titration against 200 uM CoaE. *B*, a representative isotherm of 1 mM CoA titration against 200 uM CoaE.

To explore the mechanism of formation of the ternary complex, i.e. the order of substrate binding, two sets of experiments were designed. In the first set, DCoA binding to the enzyme was studied, while in the second set, CoaE was first saturated with ATPγS and then DCoA was added as the binding ligand. Fig. 4 shows the representative titrations of DCoA against CoaE alone (A and B) and ATPγS-saturated CoaE (C and D). The mean values of the thermodynamic parameters at 20°C and pH 7.8 for the titration of DCoA against substrate-free enzyme, resulting from five independent measurements, are *K_B_* = 1.43×10^−4^ M^−1^ and Δ*H*_bind_ = −3719 cal mol^−1^ (Table 1). As can be seen from Fig. 4*A,B and B,C* there was no significant difference in DCoA binding to the enzyme in the presence or absence of ATPγS and similar values of change in enthalpy and binding constant were obtained. On the other hand, ATP (or ATPγS), by itself, did not bind to CoaE (Table 1, Fig. 4*E* and *G*). Interestingly, when ATPγS was titrated against DCoA-saturated CoaE, a good binding isotherm was observed, with *K_B_* and Δ*H*_bind_ values obtained from five independent measurements, as 2.06×10^−4^ M^−1^ and −3776 cal mol^−1^ respectively. Thus our experiments illustrate that CoaE follows a sequential mechanism of substrate addition with DCoA as the leading substrate and ATP following in tow. While the reaction product, ADP has a moderate affinity with *K_B_* and Δ*H*_bind_ values of 2.03×10^−4^ M^−1^ and −3612 cal mol^−1^ respectively, the product, CoA fails to bind to the enzyme irrespective of the presence or absence of ADP (Fig. 5*B*). Thus the CoaE reaction occurs mostly in the forward direction.

### Phosphate Donor Specificity of CoaE

**Figure 6:**
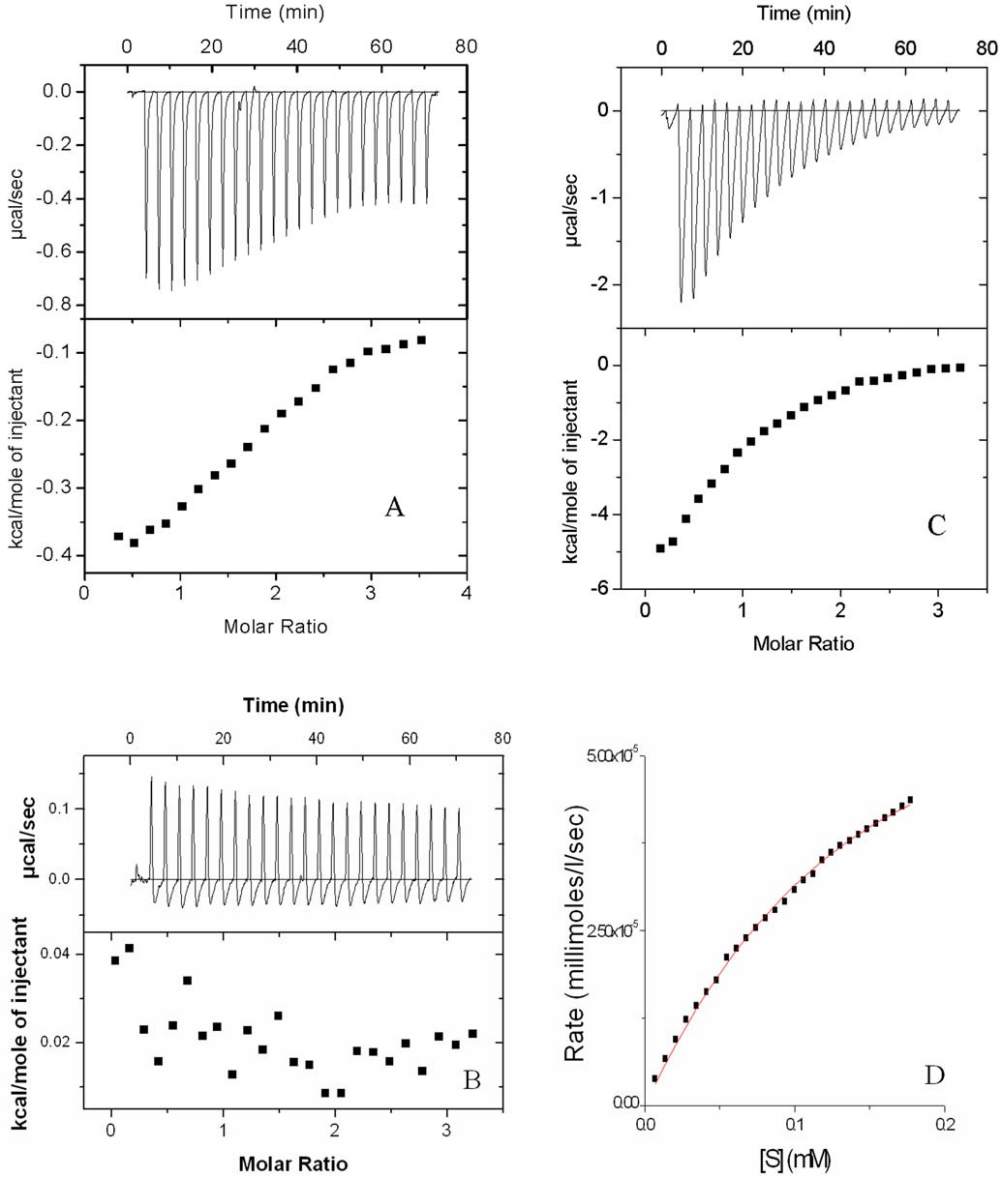
Phosphate donor specificity analysis. Binding and kinetic reactions were carried out with alternate phosphate donors. *A, B and C*, Representative binding isotherms for 1 mM dATP, 1 mM GTP and 1 mM CTP binding to 1 mM DCoA-saturated 200 uM CoaE respectively. *D*, Kinetics with 1 mM dATP. In *A, B* and *C*, the lower plot shows the integrated area under the binding curve, per experimental point.

We also determined the phosphate donor specificity of CoaE using various NTPs and dNTPs as donors. Both the assay systems gave consistent results. GTP, CTP, ADP, dATP, dGTP were used as phosphate donors and both, kinetics as well as binding energetics, were elucidated for each of them. dATP was weaker as a phosphate donor compared to ATP (Fig. 6A) and exhibited a Km of 320 uM (Fig. 6*D*). In contrast, dGTP and GTP were incapable of donating a phosphate group both in the presence and absence of DCoA and neither bound the enzyme well. The data for GTP as a representative of these is shown in Fig. 6*B*. Interestingly, CTP was inactive in phosphorylating DCoA but it bound CoaE well exhibiting a binding constant of 1.59×10^−4^ M^−1^(Fig. 6*C*) in the absence of DCoA. In contrast, CTP fails to bind to the CoaE-DCoA complex.

### Influence of CTP on CoaE

Following up on the lead of strong binding, but a lack of DCoA-phosphorylating activity of CTP, the latter's effect on CoaE activity and catalysis was further examined. The enzymatic reaction was inhibited upon preincubation with CTP, exhibiting a Vmax of 0.89 uM min^−1^, compared to a Vmax of 1.75 uM min^−1^ in the absence of CTP. On the other hand, CTP did not inhibit the reaction upon addition to a DCoA-saturated CoaE reaction.

### Homology Modeling of the Mycobacterial CoaE

Extensive computational analyses on the full length mycobacterial CoaE and its individual domains was carried out (Supporting Information File S1, Table S2).The mycobacterial enzyme was homology modeled and this structure was then validated extensively (Supporting Information File S1, Fig. S3). We subjected our modeled, energy-minimized NTD-CoaE structure to a structural homology search by the DALI server which generated 27 proteins with strong structural similarity (*Z* score >7) to the mycobacterial CoaE. All these proteins are actually structural neighbors of CoaE in the P-loop containing NTP hydrolases superfamily under SCOP [25]. Remarkably, a majority of very close structural homologs to the mycobacterial CoaE are the uridylmonophosphate/cytidylmonophosphate kinases, deoxycytidine kinases and the cytidylate kinases from several different sources, with RMSD values in the range of 2.8–3 Å and Z-scores in the ranges of 8.8–11.6 for more than an average of 148 in 194 residues. This is interesting in the light of the fact that CTP inhibits mycobacterial CoaE activity and a DALI search also picks CTP-binding proteins as close structural homologs, further lending credence to the strong binding shown by CTP to the enzyme and its role in enzyme regulation.

### Ligand Docking Analyses

**Figure 7:**
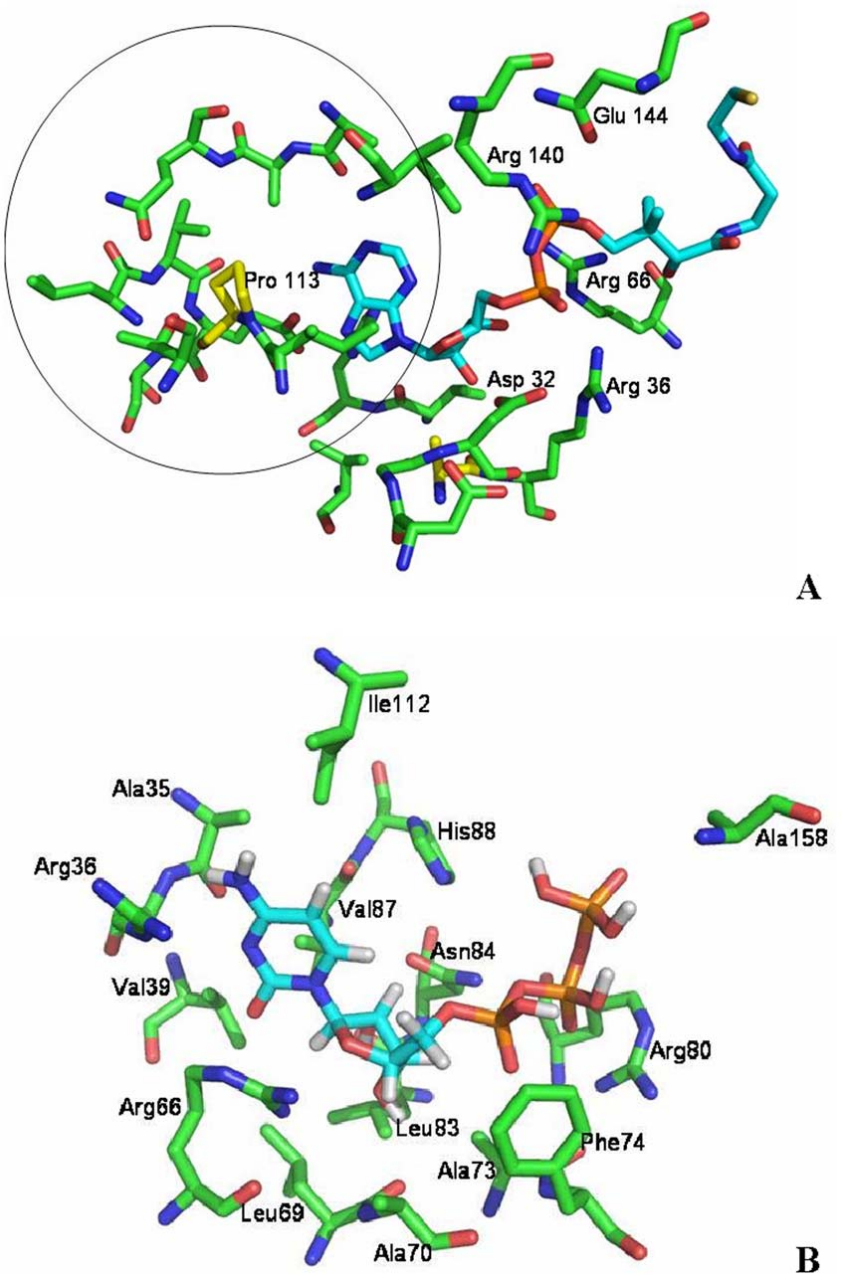
Docking studies. The protein residues have been drawn as green ball-and-stick models and the ligands in cyan. The figures were generated using PyMol. *A*, Schematic depicting the residues in the mycobacterial CoaE interacting with DCoA and the docked ligand. The encircled residues are the ones which interact with the leading substrate and differ from those of the host enzyme. *B*, Schematic depicting the residues in the mycobacterial CoaE interacting with CTP and the docked ligand.

We carried out a detailed comparative analysis of the residues in the mycobacterial CoaE interacting with the ligands, the enzyme's substrate, DCoA; its product, CoA and its metabolic regulator, CTP, calculating the distance between the interacting amino acid residues and the ligand (Å), the surface area of contact (Å^2^) and the number of contacts formed by an individual residue with the ligand (Tables 2 and 3, Supporting Information File S1). DCoA was docked onto the modeled CoaE and it fit snugly in the deep cleft between the core and CoA domains similar to the putative DCoA-binding site for the *Haemophilus* enzyme proposed by Obmolova *et al.* [26], the interaction being stabilized by 26 hydrogen bonds and 20 hydrophobic interactions (Table 2). DCoA bound the enzyme in such a way that its adenine base was in a hydrophobic environment provided by Ala35, Val87, Val91, Ile112, Leu114, Leu115, Ala157 and Ala158 (Fig. 7A) all of which are conserved residues. Glu117 and His88 make strong hydrogen bonds with the adenine base of the DCoA molecule in hydrophobic contact with the side chains of Val 91, Ile 112.

### DXD Motif

In this orientation, the 3′ OH group of the ribose is poised for interaction with the catalytic Asp32, the carboxyl group of which is within hydrogen bonding distance. The conserved Asp32 supports the coordination of Mg^2+^ and is the general base that fuels nucleophile activation for an attack on the ATP γ-phosphate. Interestingly, we observed that the catalytic aspartate exists in the sequence in tandem with another aspartate residue forming a DXD motif (where X can be any amino acid), in almost all of the dephosphocoenzyme A kinases, ranging from the extremophiles *Thermus thermophilus, Dienococcus radiodurans*; root-nodule forming *Rhizobium*; pathogens mycobacteria, corynebacteria; symbiotic lactobacilli; the worm, *C. elegans*, yeasts, plants and even the mammalian enzymes. A few members of the P-loop containing superfamily ranging from gluconate kinases, shikimate kinases, thymidylate kinases, APS kinases also possess this motif. The carboxylate group of Asp 30 is close to that of Asp32 and could in all probability be increasing the p*K*a of the catalytic aspartate priming it further for nucleophile activation, allowing it to function as a stronger base, as is the case in the *E. coli* RecA protein, an ATPase, where an aspartate residue at 4.2 Å distance from the catalytic glutamate increases the latter's p*K*a and therefore primes it for faster catalysis [27]. This aspartate residue may also be involved in coordinating the Mg^2+^ ion as in shikimate and gluconate kinases [28], [29]. Like in most kinases, Arg36, Arg66, Arg140 and Gln144 help stabilize the phosphate groups of the DCoA molecule. Pro113 which is a critical residue involved in forming the hydrophobic pocket for DCoA binding and is strictly conserved among all CoaEs from prokaryotic sources is replaced by an alanine residue at the corresponding position in the mammalian bifunctional enzymes (Fig. 7A).

### CTP Docking Analyses

ATP failed to dock close to the P-loop which is not surprising in light of our binding studies which suggest that ATP does not bind to the enzyme in the absence of DCoA. Interestingly, CTP does not dock at the P-loop (Fig. 7B). While docked at a unique site, CTP shows a tight binding interaction with the enzyme, the association being stabilized by 28 hydrogen-bonded interactions and 12 hydrophobic interactions (Table 2). Basic residues, Arg80 and Asn 84 are involved in stabilizing the negative charges of the phosphates. The phosphate oxygens form several contacts with the backbone amino groups. The cytosine base is in the hydrophobic pocket formed by residues Ala35, Ala36, Ile112, Val87 and Val39. Leu69 is also involved in hydrogen-bonding interactions with the sugar while Ala73, Leu83, Val87, highly conserved residues, provide a hydrophobic environment to the ribose (Fig. 7B). The 5′-methylene group is in Van der Waals contact with Phe74. Similar residues were found to interact with CTP in the *E. coli* CoaB structure [30]. Arg66, which is completely conserved in the entire family, forms strong hydrogen bonds with the base and the ribose sugar. Interestingly, this residue is involved in stabilizing the phosphates in the DCoA molecule. A majority of these residues which interact with CTP in our docking analysis are a part of the DCoA binding site. It's binding to a site extraneous to the P-loop explains CTP's incapacity to donate a phosphoryl group despite its strong binding to the enzyme. The strong interaction shown by CTP with the residues involved in binding and stabilizing DCoA, also explains how CTP is capable of inhibiting enzyme catalysis.

### The Role of the Domain of Unknown Function, UPF0157, in Mycobacterial CoaE

In order to determine whether the mycobacterial CoaE NTD is capable of independent expression and catalysis as the full length enzyme, in the absence of the UPF0157 domain at its C-terminus, the NTD and CTD (the UPF0157 domain) were individually cloned in the pET28a+ vector. On expression in *E. coli* BL21 (DE3) competent cells, a majority (90–98%) of the hexahistidine tagged NTD protein went into the insoluble fraction. In contrast, a major fraction of the CTD alone was expressed in the soluble form. All attempts to express the NTD alone in its soluble form, even by modification of the conditions of its induction were to no avail. Also, attempts to purify the His-tagged NTD, in the absence of the CTD, from the insoluble fraction were not fruitful. Interestingly, only when it was expressed in fusion with Glutathione-S-Transferase (GST) at its C-terminus (using the pETGEXCT vector which allows the production of C-terminal fusions of GST), NTD with full retention of its enzyme activity was found to be completely (95%) in the soluble fraction. In contrast, neither did an N-terminal fusion of GST to the NTD nor the expression of GST in trans to the NTD (on a separate plasmid co-transformed with the NTD-pET plasmid) yield any protein in the soluble fraction. The full length protein that contains both the NTD and the CTD is found completely in the soluble form with full activity. The NTD alone, on the other hand, cannot be expressed without the CTD (or GST at its C-terminus). The CTD, however, fails to assist the folding of other proteins and therefore in all probability, does not act as a general chaperone in the cell (Supporting Information File S1, Fig. S1). Thus the UPF0157 domain, i.e. the mycobacterial CTD, serves a specific role in the proper folding of the NTD as evident from its appearance in soluble form with full retention of its activity.

We next determined whether deleting a part of the CTD affects the expression of CoaE in the soluble form with a retention of its full enzymatic activity. Initially, we constructed deletes of 10, 20, 30, 40 and 50 amino acids from the C-terminus of the CTD, none of which affected full-length protein expression or activity. Focusing then on the N-terminus of the CTD, 150, 100 and 50 residues were deleted from this end of the CTD. Despite all attempts, none of these yielded protein in the soluble fraction. To further delineate the exact region in the CTD that is involved in the specific NTD-chaperoning activity, we deleted 14 and 35 residues from the beginning of the CTD, both of which expressed soluble protein. Characterization of the CTD-NΔ35 mutant revealed that it has a Kcat/Km value of 0.0362 µM^−1^ min^−1^, which is approx. 72% of the wtCoaE that contains both the NTD and the full length CTD. The mycobacterial dephosphocoenzyme A kinase therefore requires a specific unit on its C-terminus which facilitates its folding, maturation and functional expression. This role was performed by GST in the NTD-GST clone and is carried out by residues 35–50 (KIACGHKALRVDHIG) of the UPF0157 domain in the full length enzyme.

Other probable functions of the UPF0157 domain were investigated but each was ruled out (Supporting Information File S1). A possible cis-regulatory role of the CTD, in terms of the full length enzyme, by affecting catalysis by the NTD upon binding the substrates and products of the kinase was ruled out. The N-terminal 200 residues of the human bifunctional CoaDE, were recently shown to target the human enzyme to the mitochondrial outer membrane and effect activation of both the enzymatic domains by membrane phospholipids [12]. We explored the possibility of a similar activation of the mycobacterial CoaE, effected in this case by the UPF0157 domain at the C-terminus, using radiolabeled ATP and phospholipid vesicles, prepared in the laboratory, but no significant enhancement of CoaE activity was seen (Supporting Information File S1, Fig. S2). The mycobacterial CTD shows a faint homology to S-adenosyl methionine (SAM)-dependent methyltransferases in a PSI-BLAST search. All attempts to assign such an activity to UPF0157 by a methyltransferase assay, using SAM as the donor, were unsuccessful. Also, lack of heat exchange when the full length CoaE was titrated against SAM under a variety of conditions ruled out such a role. We further explored the possibility of the CTD channeling the 4′-phosphopantetheine (4′-PP) moiety from CoA as soon as it is synthesized by the mycobacterial CoaE, but the cloned UPF0157 domain did not show any phosphopantetheinyl transferase activity (Supporting Information File S1).

## Discussion

CoaE catalyses phosphoryl transfer in the last step of CoA biosynthesis and has been shown as an essential enzyme. The differences between eukaryotic and prokaryotic CoaE proteins, prompted us to characterize the kinetic mechanism of CoaE from the human pathogen *M. tuberculosis*. Its indispensable role renders it an attractive target for the development of anti-mycobacterial agents. The mechanism of the forward reaction was explored and elucidated using steady-state kinetics by coupled enzyme-spectrophotometric assays and ITC. The Km values of 34.9 µM for DCoA and 56.8 µM for ATP are the lowest, compared to those reported for this class of enzymes from bacteria. The CoaE domain of the human bifunctional CoA synthase shows a very low Km of 5.2 µM for DCoA and this high affinity of the human bifunctional enzyme for DCoA may be attributed to the merger of the CoaD and CoaE activities, which possibly helps channel the DCoA product of CoaD faster to the CoaE active site, therefore resulting in a catalytically more efficient bifunctional enzyme [5].

This study reports, for the first time, that CoaE has a sequential ordered mechanism of substrate binding with DCoA being the leading substrate and ATP following in tow as ATP binding to the enzyme is not as efficient as that when the enzyme is *a priori* saturated with DCoA while DCoA binding is unaffected by the presence or absence of ATP. A more positive change in entropy (15.9 cal M^−1^ K^−1^) is seen for DCoA binding than that seen with ATP (6.85 cal M^−1^ K^−1^). This indicates that DCoA binding facilitates ATP binding by inducing conformational changes in the enzyme and preparing the ATP-binding pocket for a snug fit.

Of all the phosphate donors evaluated for the mycobacterial enzyme, dATP binds reasonably well to CoaE with a binding constant of 1.74×10^−4^ M^−1^, though it is poor in phosphoryl transfer. In contrast, GTP and dGTP fail to bind to CoaE. ADP being structurally quite similar to ATP is capable of fitting in the ATP-binding pocket but is catalytically inactive, proving the substrate specificity of CoaE for ATP. A reduction in the catalytic efficiency with ADP can also possibly be because the nucleophiles on DCoA, which attack the ATP γ-phosphate, are in closer proximity to ATP than ADP and thus the phosphoryl transfer is more efficient in the presence of ATP, besides the intrinsic lability of the high energy β−γ phosphoryl bond.

ADP's strong binding to CoaE prompted us to study the reverse reaction, i.e. the conversion of CoA and ADP to DCoA and ATP, which we observed was extremely slow and in all probability, negligible in the cell. This was lent further credence by the fact that CoA binds extremely poorly to CoaE. Also, considering the fact that CoaE synthesizes a critical cell metabolite, it is safe to conclude that a majority of the reaction goes on in the forward direction to constantly replenish the ever-depleting CoA pool in the cell. CoA is known to feedback regulate its biosynthesis by acting on the first committed step of the pathway, catalyzed by pantothenate kinase and inhibiting the reaction [31]. The non-binding of CoA to CoaE also shows that CoA is incapable of directly feedback regulating its own production at the last step of the biosynthetic pathway.

Being structurally distant to ATP due to the pyrimidine ring, CTP's stronger binding to CoaE relative to GTP, which possesses a purine ring as ATP, came as a surprise. This raised an interesting question about the possible role of CTP in regulation of CoaE. Such a role is not uncommon in the biological context as several enzymes are regulated by NTPs other than their respective natural substrates. Therefore, we decided to further explore the relevance of its strong interaction with the mycobacterial enzyme. While CTP did not participate in phosphoryl transfer, preincubating the enzyme with CTP inhibited the enzyme. Conversely, CTP bound the enzyme poorly when the latter was preincubated with DCoA alone before the addition of CTP. Docking CTP on CoaE reveals that the there is an overlap in the binding pocket utilized by CTP and DCoA which renders an explanation for the inhibition shown by CTP. A regulatory mechanism is seen emerging from these studies whereby CTP limits the catalytic efficiency of CoaE when it binds the enzyme before the enzyme interacts with its leading substrate.

Regulation of enzyme activity by CTP is slightly unlikely in an everyday-context considering the fact that ATP is the major phosphoryl donor in the cell and is vastly abundant relative to CTP in the cellular context. The cell has possibly devised this unique mode of regulation of this critical biosynthetic enzyme to prepare itself for circumstances when ATP levels drop precipitously. Therefore, in the physiological context, this regulation might have consequence in the face of adversity, when the cell needs to minimize metabolite flow through a majority of its pathways and maintain a basal level of metabolism. Also, it has been well documented that in facultative anaerobes, like mycobacteria, the cellular CoA pools vary greatly in response to various stresses [32]–[34]. Therefore, under circumstances where the cellular ATP/CTP ratio becomes low, CTP is recruited which helps in fine-tuning the levels of the cellular CoA pool by modulating its activity, to limit the biosynthesis of CoA. Thus CTP might help in regulating CoA biosynthesis under stress conditions therefore helping the organism tide over harsh conditions. In view of the evidence presented here, the currently held view of the regulation of the CoA pathway only at its first step needs to be reconsidered with a greater role allocated to CoaE than previously assigned. Considering the ever-changing environment this robust organism faces in the host, with a regular onslaught of drugs and the need to constantly fine tune its metabolism according to the surrounding conditions, regulation effected by CTP may be an effective system to bring about rapid changes in the cellular CoA levels.

We further validated our results by modeling the mycobacterial CoaE and studying its interactions with its substrates, DCoA and ATP; its product, CoA and the intrinsic metabolic regulator, CTP. Docking studies show a snug fit for DCoA in the acceptor substrate binding site, well poised for a nucleophilic attack on the ATP γ-phosphate, its interaction being stabilized by a total of 89 contacts (Table 2). Docking also revealed residues interacting with the leading substrate in the pathogen's enzyme which are different from the ones in the human enzyme (Fig. 7B). These can therefore be exploited for developing inhibitors against the enzyme. Our docking analyses also explain the inability of CTP to serve as a phosphoryl donor to DCoA as it shows specific and stoichiometric binding at a site distinct from that of ATP (the P-loop). These results also unequivocally explain the kinetic inhibition shown by CTP by virtue of its binding at the same site as DCoA and therefore obscuring the acceptor substrate binding site.

The mycobacterial enzyme has two domains as revealed by BLAST analysis, a 196-residue NTD homologous to known dephosphocoenzyme A kinases and a 211-residue CTD belonging to a family of proteins of unknown function (UPF0157). When attempts to purify the NTD alone were not fruitful, it appeared imperative to rethink the strategy being employed. Considering the fact that the mycobacterial genome has undergone a considerable amount of downsizing with a concomitant loss of redundant genes, there has to be a rationale for the organism carrying the supposed ‘burden’ of a domain of no function, the CTD of the mycobacterial CoaE. We probed a possible role of the UPF0157 domain in assisting the NTD in folding as a separately cloned NTD alone was incapable of independent, soluble expression. Such a function is not uncommon in the biological context and has been observed in several multidomain proteins, an example being the human CoaDE bifunctional enzyme complex which possesses a third domain apart from the two enzymatic domains. This 200-residue N-terminal domain in the human enzyme complex is absolutely required for the proper expression and folding of the protein when overexpressed in *E. coli*. Thus, the CTD of the mycobacterial CoaE might also act in a similar fashion and thereby assist the NTD in attaining its biologically functional conformation. If the CTD performs such a function, replacing it with a protein of similar function should aid NTD solubilization to some extent. Indeed, consistent with this hypothesis, expression of the NTD in fusion with glutathione-S-transferase at its C-terminus led to the expression of a soluble NTD that showed 95% activity of the full length protein while an N-terminal GST fusion did not yield soluble protein. Co-transformation of GST and the NTD on separate plasmids also did not facilitate NTD folding, ruling out a possible chaperonic effect in trans. Further investigations using a series of deletion mutants revealed that the stretch of amino acids from 35–50 of the UPF0157 domain are indispensable for the proper soluble expression and appropriate folding of a completely soluble and active NTD. The CTD, therefore, might not act as a universal chaperone in the cell but it specifically helps the mycobacterial CoaE NTD domain attain a properly folded structure. Thus, of all putative roles that we have examined for the UPF0157 domain at the C-terminus of mycobacterial CoaE, the only physiologically significant role to emerge is its ability to facilitate the expression of the NTD in a folded/catalytically competent form. Such a role could also be possible in cases where this domain is found either upstream or downstream of an acetyltransferase.

In summary, our results establish a new paradigm for dephosphocoenzyme A kinases providing a framework for understanding how this class of enzymes interact with their substrates. From this work a regulatory model for the CoA pathway emerges which provides insights, into the probable mechanisms employed by the cell, to keep the metabolite flow at this until now-underexplored, though critical step in the CoA biosynthesis under check. These studies show that the end product of CoA biosynthesis, CoA, cannot feedback regulate its own production at the last step of the pathway, but the cell utilizes other subtle means to regulate metabolite flow, using an intrinsic metabolic inhibitor, CTP, which by virtue of its binding at a site, distinct from that of ATP, is unable to act as a phosphate donor but can easily inhibit the enzyme due to its binding at a site overlapping that of the acceptor substrate, DCoA as illustrated by homology modeling and docking studies. Therefore, under conditions of metabolite paucity and stress, the cell calls upon CTP to monitor the flow at the CoaE step. In view of the evidence presented in this work, the widely understood mechanism of regulation of Coenzyme A biosynthesis; which considers feedback regulation by CoA at the pantothenate kinase step of the pathway to be the sole means of metabolite flow regulation of this critical biosynthetic pathway, needs to be revised and a broader role for the last enzyme in the pathway, CoaE, needs to be considered. We have also for the first time, experimentally established a role for the UPF0157 domain from the Pfam database in acting as a very specific chaperone.

## Supporting Information

Supporting Information File S1 (0.04 MB DOC)

Figure S1 Universal Chaperone Assays for the CTD. In order to determine whether the CTD has a general chaperonic role in the cell, DTT-induced insulin aggregation assays were carried out with the CTD and CoaE as chaperones. The assay mixture with insulin (0.6 mg/mL), DTT (30 mM) was carried out in 50 mM phosphate buffer, pH 7.4 at 37°C. Varying concentrations of the CTD and CoaE were used for protection.
(4.35 MB TIF)

Figure S2 Activation of CoaE activity by phospholipids effected by the CTD. CoaE activity was assayed using 0.2 mM DCoA, 0.25 mM ATP, 0.5 uCi γP^32^-ATP, 1.5 mM DTT, 10 mM MgCl2, 150 mM Tris, pH 8.0 and varying concentrations of phospholipids vessicles (X = 10 Mm) by the assay system used in Fig. 3C.
(0.73 MB TIF)

Figure S3 Evaluation of the quality of the homology-modeled CoaE model. ProQres a neural network based approach to predict the local quality of protein structure models which uses the atom-atom contacts, residue-residue contacts, solvent accessibility surfaces, and secondary structure information to estimate model accuracy over a sliding window of nine residues, showed model accuracy scores for the minimized CoaE model mostly in the range of 0.7–1 for each consecutive window of 9 residues. The predicted model accuracy scores range from 0 (unreliable) to 1 (reliable).
(1.32 MB TIF)

Table S1 Sequences of primers used to clone the deletes of the C-terminal domain
(0.03 MB DOC)

Table S2 Prediction of the secondary structural elements of the mycobacterial CoaE, its N-terminal domain (NTD) and its C-terminal domain (CTD)
(0.03 MB DOC)
