# Supplementary material for: The Role of UPF0157 in the Folding of M. tuberculosis Dephosphocoenzyme A Kinase and the Regulation of the Latter by CTP
Source: PLoS One. 2009 Oct 30;4(10):e7645. doi: 10.1371/journal.pone.0007645 (PMC2765170; doi:10.1371/journal.pone.0007645)
Supplement: Supporting Information File S1 — (0.04 MB DOC) [file pone.0007645.s001.doc]

**SUPPORTING INFORMATION FILE S1**

**Possible functions of CTD**

*Role as a cellular Chaperone-*In order to establish the CTD as a general chaperone in the cell, well established assays like the DTT-induced aggregation of insulin, the DTT-induced aggregation of abrin and the UV-induced aggregation of β-crystallin as assay systems were carried out, but neither the full length enzyme nor the CTD could protect any of the said proteins from aggregation, ruling out a general chaperonic role for the CTD (Fig. S1).

*Regulatory role*-In order to explore a possible regulatory role of the CTD in terms of the full length enzyme, by affecting catalysis by the NTD, ITC binding reactions of the CTD with the substrates, DCoA and ATP or the products, CoA and ADP were carried out, none of which showed any significant binding. Thus the CTD does not bind any of the reaction substrates or products ruling out a possible cis-regulatory function for this domain.

*Activation by Phospholipids-*The N-terminal 200 residues of the human bifunctional CoaDE target the enzyme to the mitochondrial outer membrane and effect activation of both the enzymatic domains by membrane phospholipids (12)**.** Though the human NTD does not possess any significant sequence homology to the mycobacterial CoaE CTD, we still explored the possibility of a similar activation happening for the mycobacterial enzyme, effected in this case by the CTD. Using γP^32^-ATP, we carried out the CoaE reaction in the presence of phospholipid vesicles, prepared in the laboratory (12) and the reaction products were analyzed by descending paper chromatography. In spite of using a wide range of concentrations of the phospholipids vesicles, no significant enhancement of CoaE activity was seen, even on densitometric analysis of the autoradiogram, ruling out a possible role of the CTD in effecting phospholipid activation of CoaE activity (Fig. S2). A PSI-BLAST search showed a faint homology of the mycobacterial CTD to SAM-dependent methyltransferases and we investigated this possibility by carrying out methyltransferase assays. Neither the full length enzyme nor the CTD showed any methyltransferase activity which was also corroborated by the fact that none of the enzymes showed any significant binding to SAM. To rule out the possibility of ATP facilitating SAM binding to the enzyme, we carried out SAM binding reactions to ATP and DCoA-saturated CTD and full length proteins, but to no effect.

*Phosphopantetheinyl transfer*-To further explore the possibility of the CTD channeling the 4’-phosphopantetheine (4’-PP) moiety from Coenzyme A as soon as it is synthesized by the mycobacterial CoaE, to other enzymes we checked for phosphopantetheinyl transfer. The rationale for the experiment was the fact that DCoA is used by the Acyl Carrier Protein (ACP) subunit of multisubunit enzymes like citrate lyase, malonate decarboxylase, citramatalate lyase to generate 2’-(5’’-triphosphoribosyl)-3’-dephosphocoenzyme A and adenine (S1). This 2’-(5’’-triphosphoribosyl)-3’-dephosphocoenzyme A thus generated is acted upon by other enzymes to convert apo-ACP to holo-ACP by attaching this prosthetic group via a phosphodiester linkage to a serine residue of the enzyme. 4’-PP activates these proteins and is involved in downstream processing of CoA. We checked for such phosphopantetheinyl transferase activity by checking for the conversion of apo-ACP to holo-ACP using the full length enzyme and the CTD, using both DCoA and externally-added CoA as the 4’-PP sources. No such conversion could be obtained with the mycobacterial enzymes on a native PAGE although *E. coli* ACP-synthase showed activity.

**Computational Analyses**

No structures are currently available for dephosphocoenzyme A kinases fused to another domain. A template structure needed to be identified to facilitate homology modeling of the mycobacterial CoaE. In order to aid the selection of modeling templates for the N- and C-terminal domains of the mycobacterial CoaE, the Sequence Feature Scan tool from the Swiss-Model server which helps predict the secondary structure, presence of disordered regions and helps assign domains in the target sequence was used. Table S2 shows the putative secondary structural elements for the full-length protein, the N- and the C-terminal domains. As expected and is seen from the PSIPRED and INTERPRO scan results from the Swiss-Model Server, the mycobacterial CoaE is a predominantly an α-helical protein (S2, S3).

*Validation of the modeled CoaE structure:* While the performance of the automated SWISS-MODEL (18) pipeline in general is continuously evaluated by the EVA project, the quality of individual models can vary significantly, therefore, we extensively evaluated the model quality using different software which estimate the local quality of the predicted structure (S4). Graphical plots of Anolea mean force potential which performs energy calculations on a protein chain and is used to assess packing quality of the models, showed that a majority of the atoms were present in a favorable energy environment (S5). Similar results were obtained from the graphical plot of the GROMOS empirical force field energy (S6).The modeled CoaE structure was further verified and validated by several different programs. The What Check Structure Assessment tool which helps in protein structure and model assessment by performing structure quality checks, showed no strange interconnections (i.e. no covalent bonds were detected between molecules with non-identical chain identifiers). No errors were detected in amino acid nomenclature. The RMS Z-score for all improper dihedrals in the structure was within normal ranges. No C-terminal groups were seen present for non C-terminal residues. The bond angle variability was found to conform to the standards i.e. bond angles were found to deviate normally from the mean standard bond angles. The RMS Z-score is expected to be around 1.0 for a normally restrained data set, and this is indeed observed for very high resolution X-ray structures. More common values are around 1.55. RMS Z-score for bond angles in the modeled CoaE structure were 1.349. All of the side chains of residues that have a planar group were found to be planar within expected RMS deviations. All of the atoms that are connected to planar aromatic rings in side chains of amino-acid residues were in the plane within expected RMS deviations. Puckering amplitudes for all proline residues were within normal ranges. The Ramachandran Z-score, expressing how well the backbone conformations of all residues correspond to the known allowed areas in the Ramachandran plot was within expected ranges for well-refined structures (Ramachandran Z-score : -0.947). The score expressing how well the -1/_-2 angles of all residues correspond to the populated areas in the database is within expected ranges for well-refined structures (_-1/_-2 correlation Z-score: -0.583). Under accessibility related checks, the distribution of residue types over the inside and the outside of the protein was found to conform to the standards (inside/outside RMS Z-score: 1.149). Under the bump checks, verifications were carried out for all interatomic distances (including symmetry transformations) and no unusual contacts or van der waals overlaps were found. No short contact distances were found between any pair of atoms. Neither were any series of residues found with a bad packing environment. There were no stretches of three or more residues each having a quality control score worse than -4.0. No stretches of four or more residues each were found having a quality control Z-score worse than -1.75. None of the residues which had a normal backbone environment possessed abnormal rotamers. Validation with the program PROCHECK indicated good stereochemistry for the model. No residues were in the disallowed regions in the Ramachandran plot. All the main chain bond lengths were found within limits, so were the bond angles and planar groups. ProQres, a neural network based approach to predict the local quality of protein structure models which uses the atom-atom contacts, residue-residue contacts, solvent accessibility surfaces, and secondary structure information to estimate model accuracy over a sliding window of nine residues, showed model accuracy scores for the minimized CoaE model mostly in the range of 0.7-1 for each consecutive window of 9 residues (Fig. S3).

**SUPPORTING INFORMATION REFERENCES**

1. Hoenke, S., Wild,M. R. and Dimroth, P**.** (2000) Biosynthesis of Triphosphoribosyl-dephospho-coenzyme A, the Precursor of the Prosthetic Group of Malonate Decarboxylase Biochemistry, 39 (43), 13223–13232
2. Zdobnov E.M. and Apweiler R. (2001) InterProScan - an integration platform for the signature-recognition methods in InterPro Bioinformatics 17,847-848
3. Jones DT. (1999) Protein secondary structure prediction based on position-specific scoring matrices. J. Mol. Biol***.*** 292, 195-202
4. Koh, I. Y., V. A. Eyrich, et al. (2003). EVA: evaluation of protein structure prediction servers. Nucleic Acids Res 31(13): 3311-3315
5. Melo, F. and E. Feytmans (1998). Assessing protein structures with a non-local atomic interaction energy. J Mol Biol 277(5): 1141-1152.
6. van Gunsteren, W. F., S. R. Billeter, et al. (1996). Biomolecular Simulations: The GROMOS96 Manual and User Guide. Zürich, VdF Hochschulverlag ETHZ
