# Supplementary figures and images for: The Role of UPF0157 in the Folding of M. tuberculosis Dephosphocoenzyme A Kinase and the Regulation of the Latter by CTP
Source: PLoS One. 2009 Oct 30;4(10):e7645. doi: 10.1371/journal.pone.0007645 (PMC2765170; doi:10.1371/journal.pone.0007645)

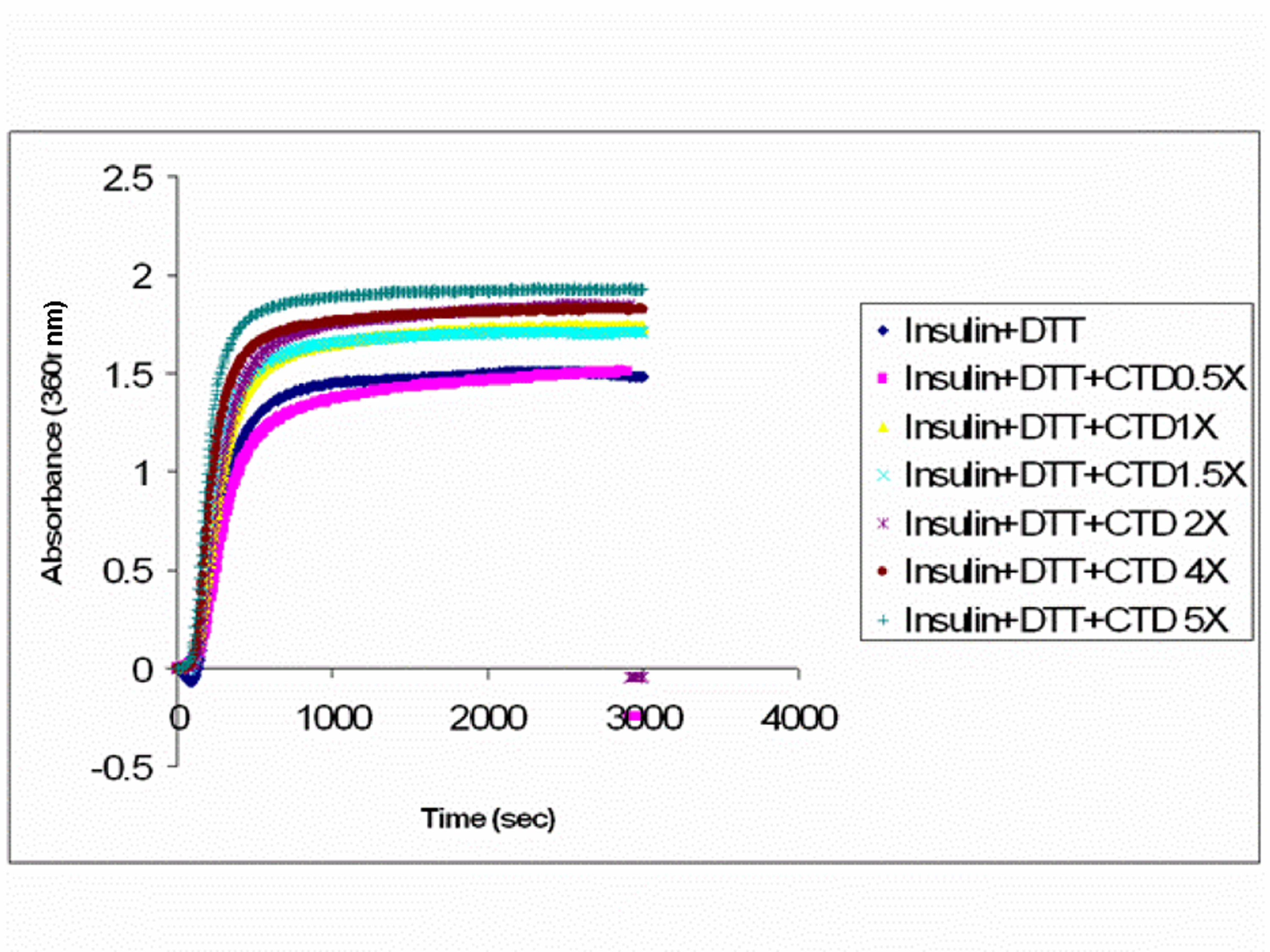

Supplement: Figure S1 — Universal Chaperone Assays for the CTD. In order to determine whether the CTD has a general chaperonic role in the cell, DTT-induced insulin aggregation assays were carried out with the CTD and CoaE as chaperones. The assay mixture with insulin (0.6 mg/mL), DTT (30 mM) was carried out in 50 mM phosphate buffer, pH 7.4 at 37°C. Varying concentrations of the CTD and CoaE were used for protection. (4.35 MB TIF) [file pone.0007645.s002.tif]

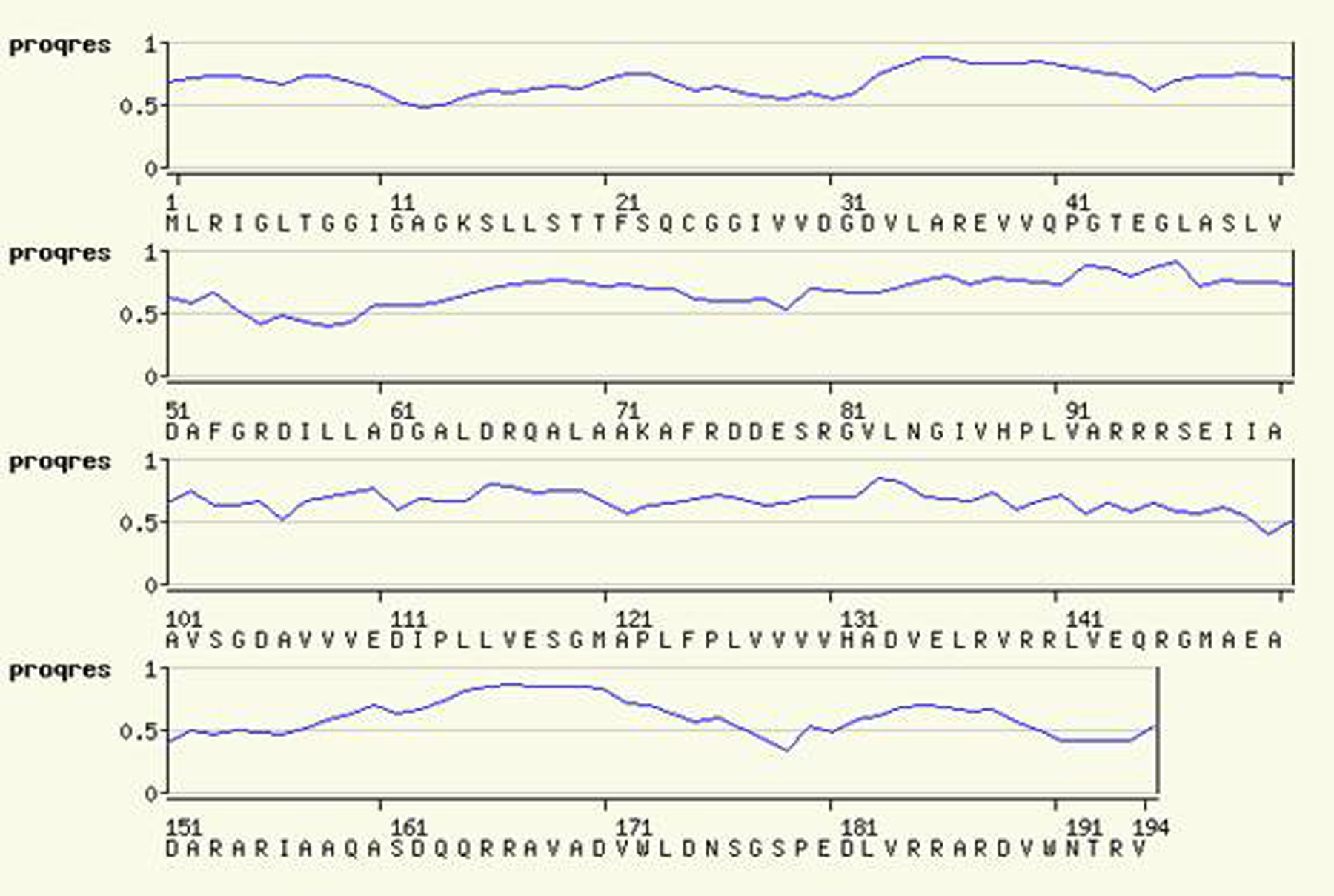

Supplement: Figure S3 — Evaluation of the quality of the homology-modeled CoaE model. ProQres a neural network based approach to predict the local quality of protein structure models which uses the atom-atom contacts, residue-residue contacts, solvent accessibility surfaces, and secondary structure information to estimate model accuracy over a sliding window of nine residues, showed model accuracy scores for the minimized CoaE model mostly in the range of 0.7–1 for each consecutive window of 9 residues. The predicted model accuracy scores range from 0 (unreliable) to 1 (reliable). (1.32 MB TIF) [file pone.0007645.s004.tif]
