# Supplementary material for: The Role of UPF0157 in the Folding of M. tuberculosis Dephosphocoenzyme A Kinase and the Regulation of the Latter by CTP
Source: PLoS One. 2009 Oct 30;4(10):e7645. doi: 10.1371/journal.pone.0007645 (PMC2765170; doi:10.1371/journal.pone.0007645)
Supplement: Table S1 — Sequences of primers used to clone the deletes of the C-terminal domain (0.03 MB DOC) [file pone.0007645.s005.doc]

| C_10_Rev | 5'-CCCAAgCTTTCACCATgCCCgCTggTAggCATC- 3' |
| --- | --- |
| C_20_Rev | 5' –CCCAAgCTTTCACggCTCCTTggCggTgACgTA- 3' |
| C_30_Rev | 5' –CCCAAgCTTTCACTCACCgTCggCgCgCCTgTC- 3' |
| C_40_Rev | 5’-CCCAAgCTTTCACTTgACCgTCAAATAgTCTTC- 3' |
| C_50_Rev | 5’ –CCCAAgCTTTCAgggATTggCCgCCAgCCAgTC- 3' |
| CTD_35_ONE | 5’ – CCgTCgATCTgACgTTgggCCAggTT- 3' |
| CTD_35_TWO | 5’- CCATCgaTcacattgggtcaaccgccgtg- 3' |
| CTD_For | 5’ –ggAATTCCATATggCgCACAA- 3’ |
| CTD_50_one | 5’ –ggAATTCAAgggCTggACgCgCgTgTTCCAgA- 3’ |
| CTD_50_two | 5’ –ggAATTCTCAACCgCCgTgTCgggCTTCC- 3’ |
| GST_NTD_Fwd | 5’ –CCCgAATTCATgCTgCgCATCgggCTgACCggC- 3’ |
| GST_NTD_Rev | 5’- CCgCTCgAgTCAACgTTgggCCAggTTgTgCgCg- 3’ |
| NTD_GEXCT_For | 5’ –CATgCCATggCAATgCTgCgCATCgggCTgACCgg- 3’ |
| NTD_GEXCT_Rev | 5’ –CgAgCTCgTCAgggACgCCAgTgCACCgCATC- 3’ |
| CTD_14_For | 5- gTTCgATCgTTgggCCAggTTgTgCgCgAA- 3’ |
| CTD_14_Rev | 5’-gATCgATCgTggCCggATCAggCgCgg- 3’ |
