# Supplementary material for: The Role of UPF0157 in the Folding of M. tuberculosis Dephosphocoenzyme A Kinase and the Regulation of the Latter by CTP
Source: PLoS One. 2009 Oct 30;4(10):e7645. doi: 10.1371/journal.pone.0007645 (PMC2765170; doi:10.1371/journal.pone.0007645)
Supplement: Table S2 — Prediction of the secondary structural elements of the mycobacterial CoaE, its N-terminal domain (NTD) and its C-terminal domain (CTD) (0.03 MB DOC) [file pone.0007645.s006.doc]

| Protein | Helix content % (residues) | β-Sheet content % (residues) | β-turn content % (residues) | Coiled coil content % (residues) |
| --- | --- | --- | --- | --- |
| CoaE | 44.5(174) | 26.1 (102) | 12 (47) | 21.5 (84) |
| NTD | 44.6 (82) | 32.6 (60) | 12.5 (23) | 19 (35) |
| CTD | 44.2 (92) | 21.2 (44) | 15.4 (32) | 26.9 (56) |
